# Is the Time Necessary to Obtain Preoperative Stabilization a Predictive Index of Outcome in Neonatal Congenital Diaphragmatic Hernia?

**DOI:** 10.1155/2012/402170

**Published:** 2012-01-04

**Authors:** Andrea Gentili, Rosina De Rose, Elisa Iannella, Maria Letizia Bacchi Reggiani, Mario Lima, Simonetta Baroncini

**Affiliations:** ^1^Department of Paediatric Anaesthesia and Intensive Care, S. Orsola-Malpighi Hospital, University of Bologna, 40183 Bologna, Italy; ^2^Department of Cardiology, S. Orsola-Malpighi Hospital, University of Bologna, 40183 Bologna, Italy; ^3^Department of Surgical Paediatrics, S. Orsola-Malpighi Hospital, University of Bologna, 40183 Bologna, Italy

## Abstract

*Background*. The study aims to verify if the time of preoperative stabilization (≤24 or >24 hours) could be predictive for the severity of clinical condition among patients affected by congenital diaphragmatic hernia. *Methods*. 55 of the 73 patients enrolled in the study achieved presurgical stabilization and underwent surgical correction. Respiratory and hemodynamic indexes, postnatal scores, the need for advanced respiratory support, the length of HFOV, tracheal intubation, PICU, and hospital stay were compared between patients reaching stabilization in ≤24 or >24 hours. *Results*. Both groups had a 100% survival rate. Neonates stabilized in ≤24 hours are more regular in the postoperative period and had an easier intensive care path; those taking >24 hours showed more complications and their care path was longer and more complex. *Conclusions*. The length of preoperative stabilization does not affect mortality, but is a valid parameter to identify difficulties in survivors' clinical pathway.

## 1. Introduction

Congenital diaphragmatic hernia (CDH) is a rare but serious disorder of the newborn, that occurs in 1/2000 to 1/5000 live births a year, and still has an elevated mortality (20–50%). The prognosis of these patients, even if it has become better than in the past, still remains unsatisfactory despite the recent acknowledgements about diagnosis, physiopathology, and treatment [1, 2].

The degree of lung hypoplasia, the persistence of pulmonary hypertension, and the rate of antenatal termination are the main factors that influence the prognosis [3]. Furthermore, in the presence of serious cardiac defects, the outcome of infants with CDH is extremely poor [4]. Associations with complex syndromes and chromosomal defects also worsen the prognosis [5].

The use of advanced respiratory support techniques, such as extracorporeal membrane oxygenation (ECMO), high-frequency oscillatory ventilation (HFOV), inhaled nitric oxide (iNO), and surfactant administration, which have been introduced over the last 10–15 years for the treatment of CDH patients, seems to have been able to improve their clinical patterns.

Important progress has also been made through the international collaboration provided by groups and institutions such as the CDH Study Group, the American Academy, and the CDH EURO Consortium [6–8]. Their, studies, involving collections of data and common reports, standardized postnatal management and followup shared by many centers, have surely done much to promote the awareness and treatment of this pathology, thereby improving diagnostic and therapeutic approaches.

In addition, many studies underline that CDH outcome might be influenced by the choice of undergoing surgical repair when a state of clinical stability has been achieved [5, 9–11].

A determining aspect in the whole clinical pathway of CDH patients, both during pregnancy and after birth, is the analysis of factors that could be predictive of outcome, with regards to both mortality and treatment complexity.

The literature proposes many studies on CDH disease severity involving the application of both prognosis-related factors measured singularly during pregnancy or at birth, or some predictive outcome models and scores [6, 12–16].

This study intends to assess whether the time needed for CDH patients to obtain preoperative stabilization (≤24 hours or >24 hours) could be predictive for the complexity of the whole clinical path of such patients.

## 2. Materials and Methods

Between January 2000 and December 2010, 77 consecutive neonates affected by CDH were treated at the Paediatric Intensive Care Unit (PICU) of our hospital.  Table 1 summarizes the main characteristics of the enrolled patients.

After obtaining written, informed parental consent, all patients were treated with the same protocol:

- continuous intravenous analgosedation with midazolam (2-3 mcg/kg/min) and fentanyl (2–4 mcg/kg/h);
- early HFOV used as the first choice and configured with mean airway pressure (MAP) between 9.5 and 14 cm H_2_O, delta P between 26 and 45 cm H_2_O, 10 Hz respiratory frequency, inspiratory time 33%, and FiO_2_ adjusted to maintain the paO_2_ between 80 and 100 mmHg;
- fluid intake between 60 and 90 mL/kg/day;
- volemic expansion with fresh frozen plasma (20–30 mL/kg/die);
- cardiac inotropic support with dobutamine and/or dopamine (5–10 mcg/kg/min);
- inhaled nitric oxide started at a dose of 20 ppm in the case of pulmonary hypertension;
- porcine-derived surfactant (70–100 mg/kg) administered if hypoxia occurred (paO_2_ < 60 mmHg with FiO_2_ > 0.8) without pulmonary hypertension associated with right-to-left shunt.

Both invasive and noninvasive cardiocirculatory monitoring (heart rate, pre- and postductal systolic, diastolic, mean blood pressure, and central venous pressure) were performed. Patients also underwent pre- and postductal arterial oxygen-saturation (SpO_2_), central body temperature, blood lactate values, and urine output. Echocardiography was made to check for cardiovascular anomalies, right and left ventricle performance, pulmonary artery pressure, and ductal shunting. Chest X-ray completed the respiratory evaluation, focusing on lung recruitment and mediastinum alignment.

CDH treatment strategy included medical stabilization before surgery, which was achieved when the patients reached and maintained for at least five hours the regularization of the following parameters, irrespective of the day of evaluation:

- five respiratory and blood-gas-derived indexes: the oxygenation index (OI) < 10, the alveolar-arterial O_2_ gradient (A-aDO_2_) < 250 mmHg, the arterial-alveolar O_2_ tension ratio (a/AO_2_) > 0.5, paCO_2_ < 55 mmHg and arterial pH > 7.35 during HFOV with FiO_2_ ≤ 0.50 and map ≤13;
- four hemodynamic and metabolic parameters: mean arterial pressure (MAP) within normal level for gestational age, absence of right-to-left ductal shunt, urine output ≥1.5 mL/kg/h, and lactate blood level below 3 mmol/L.

Only 73 patients were enrolled in the study, since 4 neonates with severe associated congenital heart diseases, characterized by intracardiac shunts, were excluded. Patent ductus arteriosus and patent foramen ovale were not classed as cardiovascular malformations.

The surgical approach was abdominal. A subcostal transverse muscle cutting incision was made on the site of the hernia, whose contents were gently reduced in the abdomen. Most diaphragmatic defects were repaired by direct sutures of the edges of the defect. A prosthetic material (1 mm Gore-Tex) was used for wide defects (>3.5 cm).

The neonates were weaned back to conventional ventilation only postoperatively, when respiratory and blood-gas-derived indexes became and remained within their normal range and FiO_2_ ≤ 0.40, Delta *P* ≤ 36 cm H_2_O, and map ≤12.

In order to fulfil the aim of the study, the CDH patients who were considered stable and underwent surgical repair were divided into two groups (patients who became stable in less than or at 24 hours versus those whose stabilization required more than 24 hours). The survival rate, the trend of respiratory and blood-gas-derived and hemodynamic indexes of medical stabilization (OI, A-aDO_2_, a/AO_2_, arterial pH and paCO_2_, MAP, absence of right-to-left ductal shunt, urine output, and lactate level) at three times (PICU admission, before surgery, and after surgery), the need for advanced respiratory support (iNO and surfactant) and other intensive care data (days of HFOV, days of tracheal intubation, length of stay in PICU and in hospital) were compared between the two groups. The difference between the two groups was also analysed using various neonatal scores: Apgar score at 1 and 5 minutes, CDHSG (CDH-Study-Group) equation at birth, SNAP II (Score for Neonatal Acute Physiology), SNAPPE II (SNAP Perinatal Extension II), PRISM III (Pediatric Risk of Mortality III), and WHSRpf (Wilford-Hall/Santa-Rosa formula).

The Apgar score at 1 and 5 minutes is determined by evaluating the newborn baby on five simple criteria on a scale from zero to two, then summing up the five values thus obtained. The resulting Apgar score ranges from zero to 10. The five criteria (Appearance, Pulse, Grimace, Activity, and Respiration) are used as a mnemonic aid. Scores 3 and below are generally regarded as critically low, 4 to 6 fairly low, and 7 to 10 generally normal [17].

The CDHSG score is generated from 2 descriptive data points (birth weight and 5-minute Apgar) within the first 5 minutes of life; the obtained score value divides the neonates into 3 groups with a predicted low risk (survival rate > 66%), intermediate risk (survival rate 34–66%), and high risk (survival rate < 33%) [6].

SNAP II measures the severity of illness in infants by utilizing physiological data collected during the first 12 hrs of care. It consists of six items, including the lowest mean blood pressure, lowest temperature, lowest pH, lowest PaO_2_/FiO_2_ ratio, urine output, and the presence of multiple seizures. SNAP II has also been modified for use as a mortality prediction model (SNAPPE II) by including birth weight, small-for-gestational age, and low Apgar score. Some dedicated tables relate the obtained score with predicted mortality [12, 18].

PRISM III is comprised of 17 clinical variables subdivided into 26 ranges that include physiological and laboratory measurements that are weighted on a logistic scale. PRISM III is an adequate indicator of mortality probability for heterogeneous patient groups in pediatric intensive care. Patients with PRISM scores of >10 are considered at high risk [19, 20].

WHSRpf formula uses blood gas values measured during the first 24 hours of life to calculate the equation: highest paO_2_— highest paCO_2_, with a cutoff value of zero or greater expected to predict survival [13].

Statistical analysis was performed using univariate logistic regression, Friedman test, Fisher exact test, and Mann-Whitney test; the null hypothesis was rejected when *P* < 0.05.

## 3. Results

A total of 77 consecutive patients were affected by CDH, with an overall survival of 72.7%. The 4 patients with associated congenital heart diseases excluded from the study presented 1 left ventricle hypoplasia, 2 Fallot tetralogies, and 1 interventricular defect.

Of the 73 patients included in the study, 55 (75.4%) reached presurgical stabilization, confirmed by the achievement of the five preestablished values of respiratory and blood-gas-derived indexes and of the four hemodynamic and metabolic parameters previously specified. 18 patients (24.6%) died before surgery, having never achieved clinical stabilization: 11 of these died within the first 24 hours, 5 within 48 hours, and 2 within 72 hours after birth. The cause of death was the suprasystemic pulmonary hypertension with right-to-left ductal shunt and the right cardiac failure associated with acute respiratory failure unresponsive to treatment.

The 55 patients underwent a surgical correction after a stabilization interval of 43.9 ± 38.7 hours (range 22–168); 33 reached clinical stability in less than or at 24 hours after PICU admission (group I), while 22 required a stabilization time of more than 24 hours (group II), with a mean of 77.8 ± 45.9 hours after PICU admission (range 30–168).

In both groups submitted to surgery the survival rate was 100%. No deaths occurred among the patients without associated congenital heart diseases, but they were considered stable and submitted to surgical repair of CDH. Survival was defined as survival to discharge from the hospital.

Table 2 shows that the respiratory and blood-gas-derived and hemodynamic index values are consistently outside the normal range at PICU admission, confirming the cardiorespiratory pattern severity for these patients. The values were normalized before surgery and after surgery in both groups. However, more stable and physiological levels can be noted in the group of patients reaching preoperative stabilization within 24 hours, in comparison to those who were stabilized after more than 24 hours. Among the considered indexes, A-aDO_2_, a/AO_2_, paCO_2_, and urine output showed a statistical significance. Another noteworthy difference between the two groups is that in group I the index values after surgery improved or remained the same as before surgery, whereas in group II the neonates tended to show a slight worsening of these indexes immediately after surgery.

In addition, all the predictive outcome models and scores analysed (Table 3) show worse values, farther outside normal ranges, and in the neonates who stabilized after 24 hours. In particular, we underline the fact that the Apgar score at 1 and 5 minutes and SNAPPE II show statistically significant differences between the two groups.

Finally, the advanced respiratory support application rates (iNO and surfactant) and certain intensive care timing indexes (days of HFO, days of tracheal intubation, and length of stay in PICU and in hospital) were significantly higher in the survivors requiring more than 24 hours for preoperative stabilization (Table 4).

## 4. Discussion

CDH shows a broad spectrum of clinical variability, thus making it very difficult to formulate a correct prognosis. At birth, in fact, one may find moderately compromised clinical conditions, or, conversely, dramatic cardiorespiratory patterns, that require immediate and highly invasive treatments that are not always successful [21, 22].

CDH is characterized by a variable degree of pulmonary hypoplasia associated with a decrease in the number of bronchial generations, alveoli, and pulmonary vessels and an increase in the muscularity of the pulmonary vascular bed. The hypoplastic lung has a small alveolar capillary membrane for gas exchange, which may be further decreased by the surfactant system dysfunction. Pulmonary capillary blood flow is decreased because of the small cross-sectional area of the pulmonary vascular bed, and flow may be further decreased by abnormal pulmonary vasoconstriction, which can be increased by a vicious circle sustained by hypoxia, hypercapnia, acidosis, and hypothermia [3, 23–25].

The clinical use of advanced respiratory assistance strategies, such as ECMO, HFOV, and iNO administration as a selective pulmonary vasodilator and surfactant administration, has surely improved CDH prognosis, but global mortality remains high, between 20 and 50%, in reported case series from all over the world [1, 2, 21, 22].

The goal to understand the best timing, according to the clinical pattern, to perform CDH surgical correction has reached a good level of consensus in the literature [5, 6, 8–11, 26–29]. The study by Nakayama et al., which demonstrated the utility of preoperative stabilization in improving respiratory compliance of CDH patients, had a fundamental role in proposing delayed surgical treatment [30]. A further step towards understanding the concept of CDH preoperative timing was achieved with subsequent studies that demonstrated how the improvement in CDH survival was due to cardiorespiratory stability more than the time between birth and surgery [31, 32]. In fact, the aim to achieve preoperative stabilization, irrespective of the time taken, is a priority in CDH treatment, since it is well known that surgical correction performed in a compensated condition can give the patient more survival chances. This is possible only with an improvement in respiratory failure (better ventilation of hypoplastic lung and recruitment of contralateral lung), and with the interruption of the right-to-left ductal shunt characteristic of suprasystemic pulmonary hypertension during the phase of CDH decompensation, in order to ameliorate hemodynamic performance [33–35].

CDH has a number of characteristic anatomical and physiopathological features, due to the neonatal age and to the specific pulmonary malformation. It could therefore be hypothesized that the use of some specific indexes and neonatal scores may be predictive of the severity and the clinical path of such pathology.

The application of predictive outcome indexes becomes particularly important in the early postnatal period, during which estimating the severity of the disease can promptly indicate the most appropriate therapies to undertake. On the basis of the achievement of specific values of five respiratory and blood-gas-derived indexes (OI, A-aDO_2_, a/AO_2_, paCO_2_, and arterial pH) and four hemodynamic and metabolic parameters (MAP, right-to-left ductal shunt absence, urine output, and lactate blood level), it was possible to determine the most opportune moment to submit the patients to surgery. The respiratory and hemodynamic indexes in question are often a commonly applied tool to assess the degree of cardiorespiratory failure in neonatal and paediatric age, but they can also well express the compensation conditions in patients affected by pathologies such as CDH [8, 36–38]. The literature shows how the failure to achieve validated indexes for the assessment of cardiorespiratory compensation in neonates affected by CDH may inhibit surgical repair [8, 29, 36, 39]. In our study, in fact, 18 patients died without having ever achieved a phase of compensation and without being submitted to surgery. On the other hand, all 55 patients who achieved preoperative stabilization, irrespective of the time taken (≤24 hours or >24 hours), presented a 100% postoperative survival, thus confirming the validity of the treatment undertaken and of the parameters chosen to define preoperative stabilization and the moment for surgical repair.

Some of the scores adopted, such as the CDHSG and WHSRpf, are specific for the assessment of outcome in CDH [6, 13]. SNAP II and SNAPPE II, which were initially validated outcome predictors in the non-CDH neonatal population, have also been reported as predictors of mortality in infants with CDH [12]. The Apgar score at 1 and 5 minutes and PRISM III are more generic scores, in that they assess respectively the newborn baby at birth and heterogeneous pediatric patient groups in NICU/PICU [17, 20]. Our data shows that the greatest part of the considered postnatal scores are reliable and concordant in CDH outcome: the values of these scores are in fact prognostically associated with a favourable outcome, in line with the complete survival registered in patients defined stable and submitted to surgery.

The present study also shows that the different duration of preoperative stabilization stage (≤24 hours or >24 hours) is indispensable for assessing the best moment in each patient to undergo surgical repair; it does not affect mortality given that all the patients considered stable survived, but can be considered a reliable index to assess the complexity and clinical severity of CDH in survivors.

The preoperative stabilization time divides the neonate survivors into two groups. The first is characterised by a normalization of the parameters within 24 hours, immediately followed by surgery. These patients show a correspondence between favourable outcome factors, good stability of the respiratory, blood-gas-derived and hemodynamic values, and a short clinical path. They belong to the category of patients that the literature on CDH reports as the most regular and the most compensated [5, 9, 11, 40]. The second group is characterized by the need for stabilization times longer than 24 hours and consequently a more delayed surgical repair. The latter patients are characterized both by more altered predictive outcome scores and by the fact that all respiratory, blood-gas-derived, hemodynamic, and metabolic indexes of clinical stabilization tend to worsen (within normal range) in the early postoperative period compared to the immediate preoperative stage and to the patients who became stabilized in the first 24 hours. In addition, these patients needed more frequent surfactant and iNO administration, and longer HFOV, tracheal intubation times, and prolonged length of PICU and hospital stay, if compared with patients needing less than 24 hours to achieve stability. Some of them probably belong to the category of patients who in the past were destined for an unfavourable prognosis but who now appear to have benefitted from the introduction of new treatment modalities and advanced respiratory support techniques [2, 41, 42].

## 5. Conclusions

Recent reports continue to confirm that CDH is a serious and severe neonatal pathology still afflicted by high mortality, that can present variable clinical pictures at birth and that requires immediate and graded steps of treatment depending on the respiratory distress and the persistence of pulmonary hypertension [8, 28, 31]. The possibility to have valid and precise indexes of reference in terms of outcome can help obtain a prompt and correct therapeutic framework.

Our study confirms the need to submit CDH patients to surgery only if they have achieved conditions of stability clearly grounded on precise parameters. Thanks to the achievement of such criteria, surgery can be performed with greater confidence and with the knowledge that a high survival rate can be expected. The study underlines that the length of preoperative stabilization does not affect mortality, but has proved a valid index in pinpointing difficulties throughout the patient's whole pathway. When the time needed for neonates to achieve stabilization is short (≤24 hours), they remain more stable in the postoperative period and have an easier and more linear intensive care path; when the time of preoperative stabilization is longer (>24 hours), the neonates are more complicated, need more intensive therapy, and have a longer and more complex care path, even after surgery.

## Figures and Tables

**Table 1:** Perinatal data of 77 patients affected by CDH.

Patient characteristics	
Male/female	49 (63.6%)/28 (36.4%)
Birth weight (gr)	2930 ± 485 (range 4300-1800)
Gestational age at delivery (wk)	37 ± 2 (range 42–32)
Term/preterm delivery	40 (52%)/37 (48%)
Left/right side	67 (87%)/10 (13%)
Inborn/outborn	71 (92.2%)/6 (7.8%)
Prenatal diagnosis	68 (88.3%)
Associated anomalies	18 (23.4%)
Associated congenital heart diseases	4 (5.2%)

**Table 2:** Trend of respiratory, blood-gas-derived, hemodynamic, and metabolic indexes at the three considered times (PICU admission, before surgery, after surgery) in the 55 patients stabilized and submitted to surgery, divided into two groups according to the duration of preoperative stabilization stage (≤24 hours or >24 hours).

	PICU admission		Before surgery		After surgery		*P*
Indexes	≤24 h	>24 h	≤24 h	>24 h	≤24 h	>24 h	
OI	12.4 ± 12.7	15.0 ± 10.5	5.6 ± 7.6	6.0 ± 4.9	4.5 ± 6.6	9.5 ± 8.9	ns
A-aDO_2_ (mmHg)	354 ± 158	369 ± 167	169 ± 122	207 ± 105	151 ± 110	220 ± 118	0.039
a/AO_2 _	0.28 ± 0.20	0.23 ± 0.18	0.54 ± 0.19	0.45 ± 0.25	0.56 ± 0.19	0.41 ± 0.23	0.047
paCO_2_ (mmHg)	46.7 ± 4.7	61.4 ± 6.3	37.4 ± 1.4	39.5 ± 1.9	36.6 ± 1.7	45.0 ± 2.3	0.015
pH	7.29 ± 0.19	7.25 ± 0.21	7.41 ± 0.12	7.44 ± 0.09	7.43 ± 0.12	7.42 ± 0.15	ns
PAM (mmHg)	41.5 ± 8.1	39.7 ± 8.7	42.3 ± 9.3	38.9 ± 7.7	42.1 ± 6.8	37.4 ± 6.7	ns
Urine output (mL/h)	1.65 ± 0.84	1.19 ± 0.78	2.14 ± 0.77	1.83 ± 0.65	1.96 ± 0.91	1.52 ± 0.76	0.048
Lactate (mmol/L)	1.8 ± 0.4	2.1 ± 0.7	1.2 ± 0.5	1.5 ± 0.4	1.4 ± 0.6	1.9 ± 0.9	ns
No R-L shunt	26/33 (79%)	4/22 (19%)	33/33 (100%)	22/22 (100%)	33/33 (100%)	20/22 (90%)	ns

**Table 3:** Comparison of severity scores in the 55 patients stabilized and submitted to surgery, divided into two groups according to the duration of preoperative stabilization stage (≤24 hours or >24 hours).

Scores	≤24 h	>24 h	*P*
APGAR 1 min	6.3 ± 1.6	5.1 ± 1.9	0.025
APGAR 5 min	7.7 ± 1.2	6.8 ± 1.7	0.033
CDHsg	75 ± 16	67 ± 13	ns
PRISM	13.22 ± 4.81	16.81 ± 8.32	ns
SNAP II	13.03 ± 9.99	19.74 ± 17.90	ns
SNAPPE II	18 ± 14	32 ± 23	0.016
WHSRpf	156 ± 109	144 ± 111	ns

**Table 4:** Comparison of advanced respiratory therapies and the timing of intensive care and hospital treatment in patients stabilized and submitted to surgery, divided into two groups according to the duration of preoperative stabilization stage (≤24 hours or > 24 hours).

Parameter	≤24 h	>24 h	*P*
iNO treatment	7/33 (21.2%)	18/22 (81.8%)	0.018
Surfactant treatment	9/31 (29.0%)	11/19 (57.8%)	0.041
HFO (days)	5.2 ± 4.6	7.4 ± 5.2	0.032
IRT (days)	21.6 ± 18.2	26.3 ± 17.5	0.228
PICU (days)	23.1 ± 13.7	34.1 ± 19.6	0.017
Hospital (days)	35.3 ± 12.1	43.1 ± 14.5	0.029

## References

[1] Stege G, Fenton A, Jaffray B (2003). Nihilism in the 1990s: the true mortality of congenital diaphragmatic hernia. *Pediatrics*.

[2] Colvin J, Bower C, Dickinson JE, Sokol J (2005). Outcomes of congenital diaphragmatic hernia: a population-based study in Western Australia. *Pediatrics*.

[3] Peetsold MG, Heij HA, Kneepkens CMF, Nagelkerke AF, Huisman J, Gemke RJBJ (2009). The long-term follow-up of patients with a congenital diaphragmatic hernia: a broad spectrum of morbidity. *Pediatric Surgery International*.

[4] Graziano JN (2005). Cardiac anomalies in patients with congenital diaphragmatic hernia and their prognosis: a report from the Congenital Diaphragmatic Hernia Study Group. *Journal of Pediatric Surgery*.

[5] Robinson PD, Fitzgerald DA (2007). Congenital diaphragmatic hernia. *Paediatric Respiratory Reviews*.

[6] Lally KP, Jaksic T, Wilson JM (2001). Estimating disease severity of congenital diaphragmatic hernia in the first 5 minutes of life. *Journal of Pediatric Surgery*.

[7] Lally KP, Engle W, American Academy of Pediatrics Section on Surgery, American Academy of Pediatrics Committee on Fetus and Newborn (2008). Postdischarge follow-up of infants with congenital diaphragmatic hernia. *Pediatrics*.

[8] Reiss I, Schaible T, van Den Hout L (2010). Standardized postnatal management of infants with congenital diaphragmatic hernia in Europe: the CDH EURO consortium consensus. *Neonatology*.

[9] Bagolan P, Casaccia G, Crescenzi F (2004). Impact of a current treatment protocol on outcome of high-risk congenital diaphragmatic hernia. *Journal of Pediatric Surgery*.

[10] Migliazza L, Bellan C, Alberti D, Auriemma A, Burgio G, Colombo GLEA (2007). Retrospective study of 111 cases of congenital diaphragmatic hernia treated with early high-frequency oscillatory ventilation and presurgical stabilization. *Journal of Pediatric Surgery*.

[11] Chiu P, Hedrick HL (2008). Postnatal management and long-term outcome for survivors with congenital diaphragmatic hernia. *Prenatal Diagnosis*.

[12] Skarsgard ED, MacNab YC, Qui Z, Little R, Lee SK (2005). Snap-II predicts mortality among infants with Congenital Diaphragmatic Hernia. *Journal of Perinatology*.

[13] Schultz CM, DiGeronimo RJ, Yoder BA (2007). Congenital diaphragmatic hernia: a simplified postnatal predictor of outcome. *Journal of Pediatric Surgery*.

[14] Baird R, MacNab YC, Skarsgard ED (2008). Mortality prediction in congenital diaphragmatic hernia. *Journal of Pediatric Surgery*.

[15] Eren S, Ciris F (2005). Diaphragmatic hernia: diagnostic approaches with review of the literature. *European Journal of Radiology*.

[16] Bohn D, Tamura M, Perrin D, Barker G, Rabinovitch M (1987). Ventilatory predictors of pulmonary hypoplasia in congenital diaphragmatic hernia, confirmed by morphologic assessment. *Journal of Pediatrics*.

[17] Apgar V (1953). A proposal for a new method of evaluation of the newborn infant. *Current Researches in Anesthesia &amp; Analgesia*.

[18] Richardson DK, Corcoran JD, Escobar GJ, Lee SK (2001). SNAP-II and SNAPPE-II: simplified newborn illness severity and mortality risk scores. *Journal of Pediatrics*.

[19] Pollack MM, Ruttimann UE, Getson PR (1988). Pediatric risk of mortality (PRISM) score. *Critical Care Medicine*.

[20] Gemke RJ, van Vught JA (2002). Scoring systems in pediatric intensive care: PRISM III versus PIM. *Intensive Care Medicine*.

[21] Brownlee EM, Howatson AG, Davis CF, Sabharwal AJ (2009). The hidden mortality of congenital diaphragmatic hernia: a 20-year review. *Journal of Pediatric Surgery*.

[22] Mah VK, Zamakhshary M, Mah DY (2009). Absolute vs relative improvements in congenital diaphragmatic hernia survival: what happened to ‘hidden mortality’. *Journal of Pediatric Surgery*.

[23] Reid LM (1984). Lung growth in health and disease. *The British Journal of Diseases of the Chest*.

[24] Beals DA, Schloo BL, Vacanti JP, Reid LM, Wilson JM (1992). Pulmonary growth and remodeling in infants with high-risk congenital diaphragmatic hernia. *Journal of Pediatric Surgery*.

[25] Zimmermann LJI, Janssen DJMT, Tibboel D, Hamvas A, Carnielli VP (2005). Surfactant metabolism in the neonate. *Biology of the Neonate*.

[26] Lally KP (2002). Congenital diaphragmatic hernia. *Current Opinion in Pediatrics*.

[27] Reyes C, Chang LK, Waffarn F, Mir H, Warden MJ, Sills J (1998). Delayed repair of congenital diaphragmatic hernia with early high- frequency oscillatory ventilation during preoperative stabilization. *Journal of Pediatric Surgery*.

[28] Bohn D (2002). Congenital diaphragmatic hernia. *American Journal of Respiratory and Critical Care Medicine*.

[29] Cacciari A, Ruggeri G, Mordenti M (2001). High-frequency oscillatory ventilation versus conventional mechanical ventilation in congenital diaphragmatic hernia. *European Journal of Pediatric Surgery*.

[30] Nakayama DK, Motoyama EK, Tagge EM (1991). Effect of preoperative stabilization on respiratory system compliance and outcome in newborn infants with congenital diaphragmatic hernia. *Journal of Pediatrics*.

[31] Rozmiarek AJ, Qureshi FG, Cassidy L, Ford HR, Hackam DJ (2004). Factors influencing survival in newborns with congenital diaphragmatic hernia: the relative role of timing of surgery. *Journal of Pediatric Surgery*.

[32] De Buys Roessingh AS, Dinh-Xuan AT (2009). Congenital diaphragmatic hernia: current status and review of the literature. *European Journal of Pediatrics*.

[33] Doyle NM, Lally KP (2004). The CDH study group and advances in the clinical care of the patient with congenital diaphragmatic hernia. *Seminars in Perinatology*.

[34] Logan JW, Cotten CM, Goldberg RN, Clark RH (2007). Mechanical ventilation strategies in the management of congenital diaphragmatic hernia. *Seminars in Pediatric Surgery*.

[35] Downard CD, Jaksic T, Garza JJ (2003). Analysis of an improved survival rate for congenital diaphragmatic hernia. *Journal of Pediatric Surgery*.

[36] Gentili A, Giuntoli L, Bacchi Reggiani ML Neonatal congenital diaphragmatic hernia: respiratory and blood-gas derived indexes in choosing surgical timing.

[37] Ventre KM (2006). Oxygenation index predicts outcome of acute hypoxemic respiratory failure. *AAP Grand Rounds*.

[38] Miguet D, Claris O, Lapillonne A, Bakr A, Chappuis JP, Salle BL (1994). Preoperative stabilization using high-frequency oscillatory ventilation in the management of congenital diaphragmatic hernia. *Critical Care Medicine*.

[39] Javid PJ, Jaksic T, Skarsgard ED, Lee S (2004). Survival rate in congenital diaphragmatic hernia: the experience of the Canadian Neonatal Network. *Journal of Pediatric Surgery*.

[40] Boloker J, Bateman DA, Wung JT, Stolar CJH (2002). Congenital diaphragmatic hernia in 120 infants treated consecutively with permissive hypercapnea/spontaneous respiration/elective repair. *Journal of Pediatric Surgery*.

[41] Sakurai Y, Azarow K, Cutz E, Messineo A, Pearl R, Bohn D (1999). Pulmonary barotrauma in congenital diaphragmatic hernia: a clinicopathological correlation. *Journal of Pediatric Surgery*.

[42] van den Hout L, Reiss I, Felix JF (2011). Risk factors for chronic lung disease and mortality in newborns with congenital diaphragmatic hernia. *Neonatology*.

